# Development, Characterization, and In Vitro Hemostatic Assessment of an Alginate-Based Wound Dressing Incorporating Liposomal Curcumin

**DOI:** 10.3390/gels12070626

**Published:** 2026-07-14

**Authors:** Florina Antonela-Loredana Nagy, Leonard Mihaly Cozmuta, Melisa Marcus, Beatrice Mihalescu, Anca Mihaly Cozmuta

**Affiliations:** Faculty of Sciences, Technical University of Cluj Napoca, Victoriei 76, 430072 Baia Mare, Romania; nagy.fl.antonela@student.utcluj.ro (F.A.-L.N.); leonard.mihaly@cb.utcluj.ro (L.M.C.); boje.ad.melisa@student.utcluj.ro (M.M.); mihalescu.va.beatric@student.utcluj.ro (B.M.)

**Keywords:** alginate wound dressing, liposomal curcumin, bioactive wound dressing, hemostasis, calcium ion release, fibrin secondary structure

## Abstract

Exuding wounds require dressings that combine effective exudate management, hemostatic activity, and bioactive therapeutic effects. Curcumin possesses antioxidant and anti-inflammatory properties, but its clinical application is limited by poor bioavailability and uncertain effects on coagulation. This study aimed to develop an alginate-based dressing incorporating liposomal curcumin and evaluate its physicochemical and in vitro hemostatic properties. Alginate dressings containing 0–0.5% liposomal curcumin were prepared and characterized. The selected 0.2% liposomal curcumin (D_0.2) formulations were evaluated for swelling behavior, dimensional stability, water vapor transmission rate, optical properties, calcium and polyphenol release, prothrombin time, fibrin morphology, and fibrin secondary structure in simulated serous, inflammatory, and infected wound exudates. D_0.2 exhibited favorable macroscopic integrity and flexibility, enhanced swelling capacity, improved exudate management, and favorable moisture-vapor permeability. The dressing showed controlled release of Ca^2+^ and polyphenols, producing a biphasic hemostatic response characterized by transient early coagulation delay followed by accelerated clot formation. Fibrin analysis revealed improved network organization and preservation of ordered protein conformations, particularly under inflammatory and infected conditions. Liposomal curcumin-modified alginate dressings combine effective fluid handling with adaptive hemostatic performance and enhanced fibrin stabilization, demonstrating promising potential for the management of serous, inflammatory, and infected wounds.

## 1. Introduction

The skin serves as the primary interface between the body and the external environment, acting as a protective barrier against physical, chemical, and biological insults. As the first line of defense, it is frequently exposed to burns, mechanical trauma, and other injuries that disrupt skin integrity and alter tissue structure and function. Wound healing is a coordinated and dynamic process comprising four overlapping phases: hemostasis, inflammation, proliferation, and tissue remodeling [1]. Hemostasis occurs immediately after injury through clot formation and platelet activation, limiting blood loss and initiating repair. The inflammatory phase involves immune cell infiltration for pathogen clearance and debris removal. It is accompanied by signaling that promotes tissue repair. However, prolonged inflammation and excessive reactive oxygen species can disrupt redox balance, leading to oxidative stress and impaired healing [2]. The proliferative phase includes granulation tissue formation, angiogenesis, collagen deposition, and re-epithelialization [3,4], while remodeling restores tissue strength through collagen reorganization and maturation [5]. Dysregulation in any phase, including impaired hemostasis or excessive inflammation, can lead to chronic non-healing wounds, highlighting the importance of agents that support early hemostasis and modulate inflammation.

Wound dressings enriched with antioxidants such as polyphenols, carotenoids, vitamins, saponins, tannins, or metal oxides have shown strong therapeutic potential [6,7]. Among them, curcumin, a low-molecular-weight polyphenol and principal curcuminoid of *Curcuma longa* (turmeric), has gained significant attention [8]. It is responsible for turmeric’s yellow color and is widely used in traditional medicine for inflammatory and wound-related conditions [9]. Curcumin exhibits anti-inflammatory, antioxidant, and antimicrobial activities [10,11,12,13]. It promotes wound healing by inhibiting lipid peroxidation while enhancing collagen synthesis and angiogenesis [14]. It modulates inflammation, proliferation, and remodeling phases [15,16,17]. However, its clinical use is limited by poor water solubility, low bioavailability, rapid metabolism, and potential toxicity at high topical doses [18]. To overcome these limitations, natural polymers such as chitin, chitosan, alginate, and cellulose derivatives, as well as synthetic polymers, have been widely explored as wound dressings [19,20,21,22,23]. Among these materials, alginate has attracted particular attention because it readily forms calcium-crosslinked hydrated networks. These networks effectively absorb wound exudate, maintain a moist environment, and enable controlled release of therapeutic agents while supporting hemostasis [24,25,26,27,28].

Curcumin-loaded nanoformulations within these systems improve solubility, stability, bioavailability, and controlled release, enhancing therapeutic efficacy. Liposomal and hyalurosomal systems containing curcumin, soy phosphatidylcholine, and sodium hyaluronate have shown biocompatibility and protection of keratinocytes against oxidative stress, with in vivo studies demonstrating reduced edema, decreased myeloperoxidase activity, and accelerated re-epithelialization [29]. Similarly, a nanocomposite hydrogel composed of curcumin-loaded methoxy poly(ethylene glycol)-block-poly(ε-caprolactone), oxidized sodium alginate, and chitosan provided sustained curcumin release. This system achieved complete wound closure by day 14 while enhancing granulation tissue formation, re-epithelialization, and collagen deposition [30]. Although most studies have focused on anti-inflammatory and antioxidant properties of curcumin, its effects on hemostasis remain poorly understood despite evidence of anti-hemostatic activity [31]. These effects may interfere with early clot formation and consequently delay progression to subsequent stages of wound healing.

In this context, the present study aimed to develop and characterize an alginate-based dressing incorporating liposomal curcumin and to evaluate its in vitro effects on hemostasis. Alginate was selected because its mannuronic and guluronic acid residues undergo ion-exchange-driven gelation upon contact with wound exudate. This process forms a moist and highly absorbent environment that supports both wound healing and hemostasis [32]. The prepared dressings were obtained as dried Ca^2+^-crosslinked alginate matrices. This in situ gelation can reduce bacterial contamination and maintain optimal wound conditions while releasing Ca^2+^ ions that support coagulation and cellular activity [31]. Its biocompatibility, biodegradability, and atraumatic removal further justify its use in moderately to heavily exuding wounds [32,33]. During application, they progressively rehydrate upon contact with wound exudate, producing a hydrated polymer network responsible for swelling, fluid absorption, and the controlled release of encapsulated curcumin.

## 2. Results and Discussion

### 2.1. Macroscopic Appearance

**Figure 1:**
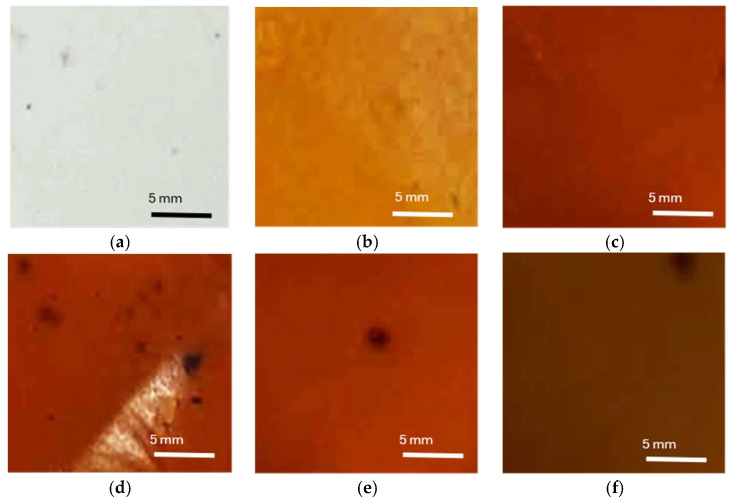
Macroscopic appearance of alginate-based dressings containing 0% (**a**), 0.1% (**b**), 0.2% (**c**), 0.3% (**d**), 0.4% (**e**), and 0.5% (**f**) liposomal curcumin.

The control dressing (Figure 1a) appeared translucent, continuous, and flexible, with no visible macroscopic defects such as cracks or fractures.

The film maintained its integrity throughout handling and exhibited no visible macroscopic cracks, fractures, or phase-separated domains. These observations indicate the successful formation of a continuous film. The relatively uniform transparency of the matrix indicates the absence of visible macroscopic heterogeneity. Overall, the D_0 dressing exhibited favorable macroscopic characteristics, including flexibility, structural integrity, and a visually uniform appearance, which are desirable properties for wound-dressing applications. The D_0.1 dressing (Figure 1b) appeared semi-transparent with an amber-golden coloration and showed a visually uniform appearance at the macroscopic level. No visible crystalline agglomerates, large macroscopic aggregates, or phase separation were observed.

The D_0.2 dressing (Figure 1c) appeared flexible, continuous, and cohesive, without visible macroscopic wrinkles, cracks, pores, or phase-separated regions. The absence of visible macroscopic aggregates indicates that no large-scale phase separation occurred during film preparation, suggesting favorable physicochemical compatibility and interfacial interaction between the bioactive compounds and the host polymer matrix.

The D_0.3 dressing (Figure 1d) exhibited distinct macroscopic and structural alterations and appeared as a dark amber-orange, semi-opaque matrix. The film showed noticeable surface irregularities, localized phase separation, and numerous dark particulate domains distributed throughout the matrix. In addition, the dressing lost its flexibility and became increasingly rigid and brittle.

The D_0.4 dressing (Figure 1e) displays a deep, translucent amber-orange coloration, exhibiting irregular boundaries, high macroscopic rigidity, and a glassy, brittle texture rather than the elastomeric flexibility required for topical medical applications.

The D_0.5 dressing (Figure 1f) exhibited a deeper and more opaque orange-amber color than the lower-concentration formulations, indicating a higher concentration of liposomal curcumin within the biopolymer network. At the macroscopic level, the dressing remained highly rigid, glassy, and brittle. Consequently, it lacked the flexibility required to conform to the wound surface.

The flexibility of the alginate dressing is provided by glycerol, which acts as a hydrophilic plasticizer between the β-D-mannuronic and α-L-guluronic acid chains. Glycerol disrupts interchain hydrogen bonding, thereby increasing the free volume and mobility of the polymer chains. However, increasing the concentration of liposomal curcumin made the composite dressings progressively more heterogeneous, rigid, and brittle. Because liposomal curcumin is highly hydrophobic, it has limited compatibility with the hydrophilic alginate–glycerol matrix. Excess liposomal curcumin therefore formed phase-separated micro-aggregates instead of being uniformly dispersed. These hydrophobic domains acted as rigid fillers that disrupted the continuity of the polymer network, restricted chain mobility, and promoted crack initiation during mechanical deformation.

Based on these preliminary macroscopic observations, the 0.2% liposomal curcumin formulation (D_0.2) was selected for further characterization. This formulation provided the best combination of visual uniformity and flexibility among the tested samples. It also exhibited the compliance required to conform effectively to anatomical surfaces. For comparative purposes, the alginate dressing without liposomal curcumin (D_0) was subjected to the identical characterization protocol to serve as a control.

### 2.2. Microscopic Morphology of Selected Dressings

**Figure 2:**
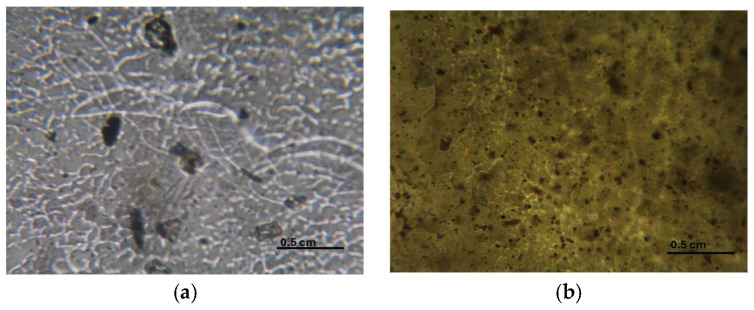
Microscopic morphology of alginate-based dressings containing 0% (**a**) and 0.2% (**b**) liposomal curcumin.

The optical micrograph of the D_0 dressing (Figure 2a) reveals a continuous, highly textured polymeric matrix characterized by a dense network of interconnected, micro-scale fibrillar and granular features.

The optical micrograph of the control dressing (Figure 2a) shows a continuous polymeric matrix with fine ridges and shallow microfolds distributed throughout the observed field. No large crystalline aggregates, cracks, or obvious phase-separated domains were observed. Overall, the optical micrograph indicates the formation of a continuous polymeric matrix prior to incorporation of the bioactive agent.

The optical micrograph of the D_0.2 dressing (Figure 2b) reveals a modified microstructure compared with the control dressing. The matrix exhibits a characteristic greenish-yellow hue consistent with the presence of liposomal curcumin while maintaining a continuous polymeric appearance with fine microfolds and shallow ridges. Dark particulate inclusions are observed throughout the field of view, consistent with the presence of liposomal curcumin-containing domains. No obvious microstructural discontinuities, large aggregates, or phase-separated regions were detected within the examined area. These observations indicate that incorporation of 0.2% liposomal curcumin did not visibly disrupt the continuity of the polymeric matrix under optical microscopy.

### 2.3. Swelling Behavior of Selected Dressings

**Figure 3:**
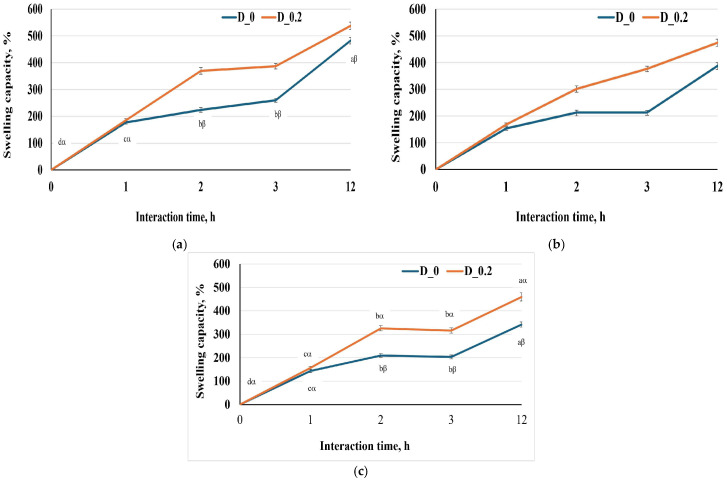
Swelling capacity of alginate dressings without (D_0) and with 0.2% liposomal curcumin (D_0.2) following immersion in serous exudate (**a**), inflammatory exudate (**b**), and infected exudate with abundant secretion (**c**). Data are presented as mean ± standard deviation (SD) (n = 3). Values marked with different letters (a, b, c, and d) for the same dressing and wound exudate simulant type are significantly different. Values marked with different letters (α and β) at the same interaction time and different wound exudate simulants are significantly different (one-way ANOVA, Tukey’s test, *p* < 0.05).

The swelling behavior of both D_0 and D_0.2 dressings was strongly time- and exudate-dependent, with a progressive increase up to 12 h under all tested conditions (Figure 3). In serous exudate (SE), the control dressing increased from 176.74% at 1 h to 482.26% at 12 h. The D_0.2 formulation exhibited consistently higher swelling after the initial time point, reaching 537.86% at 12 h. A similar trend was observed in inflammatory exudate (IE), where swelling increased from 153.17% to 388.31% for the D_0 dressing and to 474.64% for the D_0.2 dressing. In infected exudate (IASE), the control increased from 143.61% to 342.12%, whereas the D_0.2 formulation reached 459.69% at 12 h. The swelling behavior observed for D_0 and D_0.2 is consistent with previous reports showing that alginate-based wound dressings can absorb several times their dry weight due to the hydrophilic nature of alginate and its ionizable carboxyl groups, with swelling strongly dependent on calcium crosslink density and ionic composition of the surrounding medium [34]. The enhanced swelling observed in the presence of 0.2% liposomal curcumin suggests that incorporation of the lipid–polyphenol system modifies the internal architecture of the alginate network. Alginate hydrogels swell primarily through water uptake driven by ion exchange (e.g., displacement of Ca^2+^ by monovalent ions in the external medium) and relaxation of the guluronic acid–rich “egg-box” junction zones responsible for ionic crosslinking [32].

The higher swelling capacity of the liposomal curcumin-loaded system, therefore, likely reflects a reduction in effective crosslink density or increased network heterogeneity. These structural changes enlarge the mesh size of the hydrogel and facilitate fluid penetration. Liposomal curcumin may also contribute by introducing dispersed phospholipid bilayer domains into the hydrogel matrix. These domains act as structural discontinuities that disturb polymer chain packing, weaken alginate–alginate interactions, and promote water uptake [35]. Liposomes embedded in hydrogels have previously been reported to increase swelling and modify diffusion pathways due to their amphiphilic nature and steric effects within polymer networks [35]. At the molecular level, curcumin contains phenolic hydroxyl groups and a β-diketone system capable of hydrogen bonding with alginate carboxylate and hydroxyl groups. These interactions can locally increase hydration and promote water retention within the polymer matrix [36]. However, due to its high hydrophobicity and poor aqueous solubility, liposomal curcumin is predominantly partitioned into the lipid bilayer of liposomes rather than directly interacting with the hydrophilic alginate phase, which may indirectly influence polymer organization by introducing steric hindrance and altering chain packing density [35].

Comparatively, among exudates, both formulations followed the same trend (SE > IE > IASE) at intermediate and late time points, indicating that serous exudate promoted the highest fluid uptake, whereas infected exudate resulted in the lowest swelling. This behavior can be attributed to differences in composition and viscosity, as protein-rich and more complex exudates (IE and IASE) may hinder water diffusion and reduce polymer relaxation compared to less concentrated serous fluid. All systems exhibited a rapid initial swelling phase within the first 2–3 h, followed by a gradual approach to equilibrium at 12 h, consistent with a diffusion-controlled mechanism.

From a clinical perspective, the enhanced swelling capacity of the D_0.2 dressing may improve the management of moderate to highly exuding wounds. It enhances exudate sequestration and reduces fluid accumulation at the wound–dressing interface, thereby minimizing periwound maceration. The sustained uptake observed up to 12 h also suggests potential for extended wear time, which could reduce dressing change frequency and improve patient comfort. However, the higher swelling in the D-0.2 formulation should be balanced against potential risks of excessive expansion in confined wound spaces, highlighting the need for appropriate wound selection. Overall, both formulations demonstrate suitable exudate-responsive behavior, with the D_0.2 dressing showing improved fluid handling performance without altering the fundamental swelling kinetics across different wound-like environments.

### 2.4. Dimensional Stability of the Dressings

**Figure 4:**
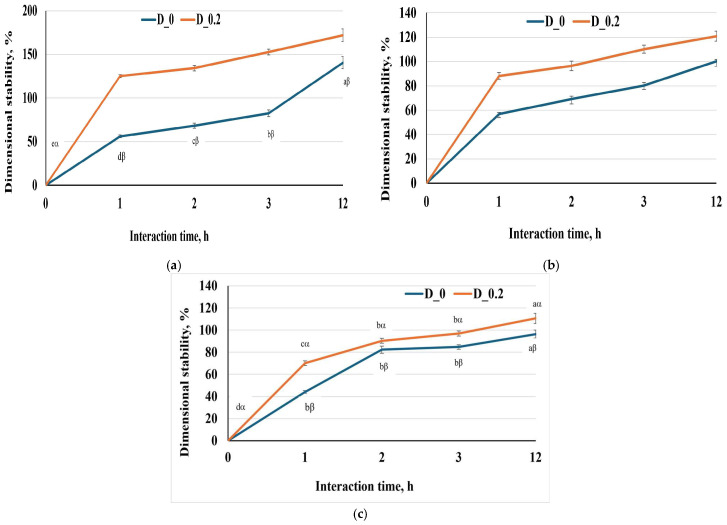
Dimensional stability of alginate dressings without (D_0) and with 0.2% liposomal curcumin (D_0.2) following immersion in serous exudate (**a**), inflammatory exudate (**b**), and infected exudate with abundant secretion (**c**). Data are presented as mean ± standard deviation (SD) (n = 3). Values marked with different letters (a, b, c, d and e) for the same dressing and wound exudate simulant type are significantly different. Values marked with different letters (α and β) at the same interaction time and different wound exudate simulants are significantly different (one-way ANOVA, Tukey’s test, *p* < 0.05).

The areal swelling ratio of both D_0 and D_0.1 dressings increased progressively over time in all tested simulated exudate environments, confirming a time-dependent expansion of the material in two-dimensional dimensions (Figure 4). In serous exudate (SE), the D-0 dressing increased from 56.10% at 1 h to 140.51% at 12 h, whereas the D_0.2 dressing exhibited markedly higher values at all time points, reaching 172.04% at 12 h. A similar trend was observed in inflammatory exudate (IE), where the control dressing increased from 56.88 to 100.26, while the D_0.2 formulation reached 120.81% at 12 h. In IASE, the control dressing rose from 44.12% to 96.41%, whereas the D-0.2 dressing showed enhanced expansion up to 110.61% at 12 h. Overall, incorporation of 0.2% liposomal curcumin consistently increased the areal swelling ratio across all exudate types and time points, indicating that it modifies the structural response of the alginate network and promotes greater planar expansion upon hydration. The time-dependent increase in areal swelling observed for D_0 and D_0.2 agrees with Boateng et al. [33], who reported progressive fluid uptake and expansion of alginate wound dressings driven by ion exchange and network relaxation in simulated wound environments.

Comparing the exudates, all formulations followed a similar trend, with SE producing the highest areal swelling, followed by IE and then IASE. This suggests that serous exudate promotes more pronounced lateral expansion, likely due to its lower protein content and viscosity, which facilitates fluid diffusion into the polymer matrix. In contrast, the reduced swelling in IE and IASE may result from higher protein content, increased ionic strength, and greater viscosity, which restrict polymer chain relaxation and limit expansion.

The enhanced swelling observed in D_0.2 dressings can be attributed to physicochemical interactions between curcumin and the alginate network. Curcuminoids may act as a structural modifier, increasing the free volume between polymer chains or slightly weakening intermolecular interactions (e.g., hydrogen bonding or ionic crosslinking). This network loosening enhances chain mobility upon hydration, allowing greater planar expansion and higher areal swelling ratios compared to the control. Curcumin liposomes may also introduce microstructural heterogeneities, creating additional pathways for water diffusion and acting as localized swelling centers that accelerate initial hydration. Differences in swelling among exudates can also be explained by ionic interactions and osmotic effects. Alginate hydrogels are sensitive to the surrounding medium’s ionic composition, with swelling governed by the interplay between osmotic pressure and ionic crosslinking, particularly involving divalent cations such as calcium. In serous exudate, low ionic strength and protein content favor osmotic swelling and freer polymer chain extension. Conversely, inflammatory and infected exudates, with higher ion concentrations, especially multivalent cations, reinforce crosslinks within the alginate network, tightening the structure and limiting expansion. Proteins may adsorb onto the polymer surface or penetrate the matrix, creating additional constraints, while higher viscosity slows water diffusion, further reducing both the rate and extent of swelling. Lower viscosity in SE allows faster and more uniform hydration, supporting greater areal expansion. Curcumin liposomes may additionally exert mild plasticizing effects, reducing the stiffness of the alginate film and facilitating deformation during hydration. Mechanistically, swelling kinetics in all systems exhibit a rapid initial phase followed by a plateau, consistent with Fickian or quasi-Fickian diffusion. Early swelling is driven by solvent penetration, while later stages reflect a balance between osmotic forces and elastic retraction of the polymer network. Curcumin liposomes enhance swelling without altering the fundamental mechanism by increasing network flexibility and hydration capacity. All systems show rapid expansion within 1–3 h, followed by a gradual increase up to 12 h, consistent with diffusion-driven swelling coupled with progressive network relaxation.

From a clinical perspective, the enhanced swelling capacity of the D_0.2 dressing may improve the management of moderate-to-highly exuding wounds. Increased swelling enhances exudate sequestration and reduces fluid accumulation at the wound–dressing interface, thereby minimizing periwound maceration. The sustained fluid uptake observed over 12 h also suggests the potential for extended wear time, which may reduce dressing replacement frequency and improve patient comfort. Nevertheless, excessive swelling should be considered when treating confined wound spaces, emphasizing the importance of selecting the dressing according to wound characteristics. Overall, the results suggest that 0.2% liposomal curcumin incorporation enhances the dimensional responsiveness of alginate dressings without altering their fundamental swelling kinetics, supporting their potential application in exuding wound environments with varying simulated wound exudate compositions.

### 2.5. Water Vapor Transmission Rate

**Figure 5:**
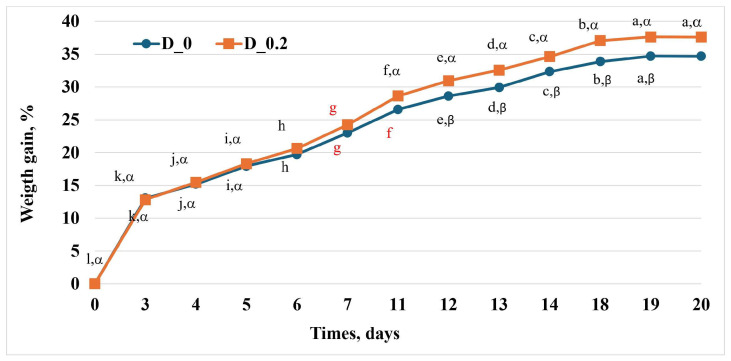
Water vapor transmission rate of alginate-based dressings containing 0% (D_0) and 0.2% (D_0.2) liposomal curcumin. Values marked with different letters a–l within the same dressing are significantly different. Values marked with different letters α and β between different dressings are significantly different (one-way ANOVA, Tukey’s test, *p* < 0.05).

The water vapor transmission rate (WVTR) of both dressings followed a time-dependent increase, reaching a plateau at later stages (Figure 5), a behavior consistent with moisture-permeable hydrogel and alginate wound dressings that regulate vapor transport until equilibrium between evaporation and matrix hydration is established [33].

The similar WVTR profiles observed for D_0 and D_0.2 up to 168 h are consistent with the dominant influence of the Ca^2+^-crosslinked “egg-box” network on vapor transport in alginate systems, as previously described for ionically crosslinked polysaccharide films where permeability is primarily governed by network density and water content. The divergence observed at later time points, with higher WVTR for D_0.2, may be associated with microstructural loosening and increased free volume induced by the incorporation of lipid-based vesicles, which has been shown in composite hydrogel systems to enhance vapor diffusion by disrupting polymer packing and increasing porosity. From a functional perspective, such modulation of WVTR may influence both ionic transport and bioactive release, since increased water-vapor exchange is often correlated with enhanced fluid mobility within hydrogel networks and accelerated diffusion of encapsulated compounds [33]. Consequently, higher WVTR in D_0.2 may simultaneously facilitate Ca^2+^ exchange with the external medium, supporting coagulation cascade activation, while also increasing diffusional fluxes that could modulate local protein–factor interactions and the release kinetics of curcumin from the dressing matrix.

### 2.6. Reflectance Spectra and Chromatic Analysis

**Figure 6:**
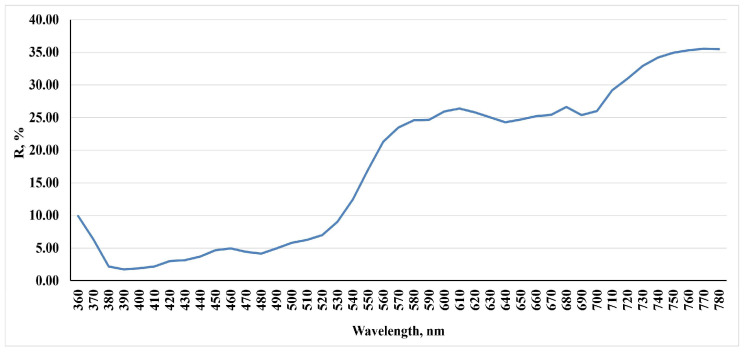
Reflectance spectra of the alginate-based dressing containing 0.2% liposomal curcumin.

The reflectance spectrum of the dressing containing 0.2% liposomal curcumin is shown in Figure 6.

At 360 nm, reflectance was approximately 10%, followed by a sharp decrease to nearly 2% in the 390–400 nm range. This region corresponds to strong absorption in the ultraviolet and blue spectral range. The absorption is attributed to curcumin encapsulated within liposomal domains distributed throughout the alginate matrix.

Curcumin is a polyphenolic compound with an extended conjugated π-electron system. This structure enables intense π→π* electronic transitions that are responsible for strong absorption in the UV–visible region [36]. Following the absorption minimum, reflectance gradually increased between 400 and 520 nm, reaching approximately 7%. A more pronounced rise was observed between 530 and 560 nm, where reflectance increased from approximately 9% to over 20%. This transition reflects two simultaneous processes. Curcumin absorption decreases in the green spectral region, while diffuse scattering from the hydrated porous alginate network increases. Beyond 600 nm, the spectrum reached a relatively stable high-reflectance plateau, with values ranging from approximately 24% to 35% between 600 and 780 nm, indicating minimal absorption in the red and near-infrared regions.

The optical behavior of the system is governed by both the intrinsic absorption of curcumin and the structural characteristics of the composite dressing. Curcumin is predominantly localized within the phospholipid bilayer of liposomes rather than being freely dispersed throughout the hydrogel. Consequently, its optical contribution arises from discrete lipid-based microdomains embedded within the alginate matrix. Micro-encapsulation does not alter the fundamental chromophoric properties of curcumin. However, it may slightly modify the spectral response because of changes in local polarity and reduced molecular aggregation. In addition to absorption effects, the alginate hydrogel contributes significantly to light scattering due to its hydrated, porous, and heterogeneous structure. The broad sigmoidal reflectance profile results from the combined effects of light absorption by liposomal curcumin and Mie-type scattering from the polymer matrix [37]. The reflectance spectrum of the D_0.2 dressing demonstrates optical characteristics potentially relevant for wound healing applications. The marked reduction in reflectance within the ultraviolet and blue regions (360–450 nm) indicates efficient attenuation of high-energy radiation [38,39]. In contrast, the higher reflectance observed in the red and near-infrared regions (600–780 nm) suggests relatively low absorption of wavelengths associated with photobiomodulation processes [40]. These wavelengths have been reported to stimulate fibroblast activity, collagen synthesis, angiogenesis, and tissue regeneration. Furthermore, the diffuse scattering provided by the alginate matrix may enhance light redistribution across the wound surface, promoting more uniform optical exposure. Overall, the D_0.2 dressing combines UV–blue shielding with the intrinsic antioxidant activity of curcumin. In addition, it exhibits favorable transmission and scattering characteristics in the red and near-infrared regions. Together, these properties support wound healing and tissue repair.

Chromatic analysis of the D_0.2 dressing (Table S1) revealed wavelength-dependent color distribution consistent with the measured reflectance behavior. The chromatic components increased progressively from the shorter to longer wavelength regions, with values of 3.87, 4.54, and 4.97 recorded in the purple (380–449 nm), blue (450–484 nm), and cyan (485–499 nm) regions, respectively. The relatively low chromatic contribution within these regions is associated with strong absorption of short-wavelength light by curcumin. In contrast, substantially higher chromatic values were observed in the green, yellow, orange, and red regions, reaching 11.26, 23.50, 25.68, and 28.39, respectively. The dominance of yellow, orange, and red components confirms the characteristic optical signature of curcumin-containing materials and explains the warm yellow-orange appearance of the dressing. The enhanced chromatic intensity at longer wavelengths is consistent with the increased reflectance observed in the visible red and near-infrared regions. This behavior indicates reduced light absorption and greater diffuse reflection within the alginate matrix. From a wound-healing perspective, the suppression of high-energy blue-violet light together with enhanced reflection in the red spectral region may be advantageous, as red wavelengths are associated with improved cellular proliferation, angiogenesis, and tissue regeneration. These findings demonstrate that the incorporation of liposomal curcumin significantly modifies the optical and chromatic properties of the alginate dressing. Such modifications may contribute to both photoprotective and therapeutic functions.

### 2.7. In Vitro Hemostatic Evaluation of Selected Dressings

**Figure 7:**
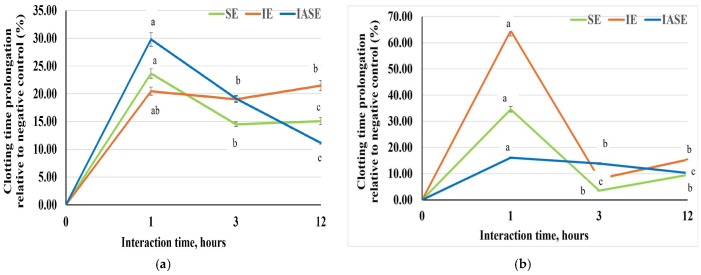
Prolongation of clotting time relative to the negative control for alginate-based dressings containing 0% (D_0) (**a**) and 0.2% (D_0.2) (**b**) liposomal curcumin in the prothrombin time assay. SE is serous exudate, IE is inflammatory exudate, and IASE is infected exudate with abundant secretion. Data are presented as mean ± standard deviation (SD) (n = 3). Values marked with different letters (a, b, c) for the same wound exudate simulant type are significantly different. (one-way ANOVA, Tukey’s test, *p* < 0.05).

The temporal prothrombin time (PT) profiles in SE (Figure 7) revealed a dynamic biphasic coagulation pattern governed by alginate matrix hydration, ion exchange, and bioactive compound release. At 1 h, both formulations prolonged coagulation relative to the negative control (D_0 = 23.65%; D_0.2 = 34.60%). Because the dressings were pre-crosslinked in a CaCl_2_ bath, Ca^2+^ ions were initially immobilized within the stable alginate “egg-box” network and therefore could not participate as free coagulation cofactors (Factor IV). Rapid hydration of the alginate matrix may also release trace soluble polysaccharide fragments, increasing the local microviscosity and sterically hindering interactions between clotting factors. In D_0.2, this initial delay may be further enhanced by the release of curcumin, which can modulate coagulation through secondary interactions with proteins involved in the coagulation cascade. By 3 h, the coagulation profile changed markedly. The PT increase for D_0.2 decreased sharply to 3.50%, significantly lower than that observed for D_0 (14.50%). This acceleration was primarily associated with Na^+^/Ca^2+^ ion exchange within the alginate matrix, whereas curcumin likely contributed only indirectly through modifications of the hydrated matrix structure. Incorporation of liposomal curcumin may alter the microstructure and local cross-linking density of the alginate network during gel formation. Upon swelling, these structural changes facilitate matrix relaxation and promote localized Ca^2+^ release. The increased availability of Ca^2+^ (Factor IV) enhances prothrombinase complex formation and shifts the system toward a predominantly procoagulant response, despite the potential anticoagulant activity of curcumin.

In IE, both dressings also prolonged coagulation at 1 h compared with the negative control. The delay was particularly pronounced for D_0.2, reaching 64.56%. This response may reflect an initial burst release of curcumin, which has been reported to inhibit serine proteases such as thrombin and Factor Xa. The burst release is likely enhanced in the inflammatory environment, where pH and ionic strength promote rapid matrix wetting and partial erosion of the outer hydrogel layer.

The PT profiles obtained in IASE exhibited a different kinetic pattern dominated by rapid matrix hydration and accelerated ion exchange. At 1 h, both formulations prolonged clotting relative to the negative control (D_0 = 29.79%; D_0.2 = 16.08%). Unlike the other simulants, D_0.2 produced a smaller initial PT prolongation than D_0. In the highly exuding infected environment, the large fluid volume and complex biochemical composition accelerate matrix hydration and initiate earlier Na^+^/Ca^2+^ ion exchange. Consequently, free Ca^2+^ ions are released sooner and partially counteract the anticoagulant effect of curcumin. By 3 h, PT values decreased for both dressings, with D_0.2 maintaining the faster clotting profile. This response is associated with progressive displacement of structural Ca^2+^ ions by Na^+^ ions from the exudate and continuous adaptation of the hydrated alginate network. Liposomal curcumin modifies the matrix microstructure and reduces the local cross-linking density, thereby facilitating matrix relaxation during swelling. Although these structural changes promote Ca^2+^ release, the improved hemostatic response cannot be attributed solely to the total amount of calcium released. Local interactions at the dressing–exudate interface and other matrix–exudate phenomena are also likely to contribute to coagulation. After 12 h, the system reached thermodynamic equilibrium, demonstrating that the D_0.2 dressing successfully adapted to highly exuding infected conditions while maintaining a balanced matrix-mediated procoagulant response.

Comparison of the three wound simulants demonstrated that the surrounding pathological environment strongly influenced the biphasic coagulation behavior of the dressings. At 1 h, SE and IE exhibited prolonged clotting that was consistent with the initial burst release of curcumin. In contrast, IASE displayed an opposite trend because rapid matrix hydration accelerated Na^+^/Ca^2+^ ion exchange. By 3 h, all three simulants showed a similar transition toward faster coagulation. PT values for D_0.2 decreased to 3.50% in SE, 8.06% in IE, and 13.89% in IASE. These findings suggest that incorporation of liposomal curcumin modifies the alginate network in a manner that facilitates the transition toward a procoagulant state. In SE and IE, this effect correlated with enhanced Ca^2+^ release. In IASE, however, the improved coagulation response appears to involve additional mechanisms beyond the total amount of calcium released. The equilibrium reached after 12 h confirms that, despite differences in early-release kinetics, all dressings ultimately developed a stable matrix-mediated procoagulant response.

The work of Maboudi et al. [41] demonstrated that incorporating curcumin into hydrophilic hydrogel matrices, such as poly(vinyl alcohol)/hyaluronic acid networks, does not significantly alter coagulation behavior. No meaningful prolongation of clotting time was observed, even at a 2% curcumin loading. These findings suggest that the hydrophilic hydrogel environment limits the immediate bioavailability of curcumin to clotting factors, thereby reducing its influence on prothrombin-mediated coagulation.

### 2.8. Release Kinetics of Bioactive Compounds

For the D_0 dressing, the hemostatic response can be primarily attributed to calcium ion release from the hydrogel network, as alginate does not contribute significant polyphenolic content. In contrast, for the D_0.2 dressing, the presence of released curcumin should be considered, as these may interact with coagulation pathways and modulate the overall hemostatic effect.

**Figure 8:**
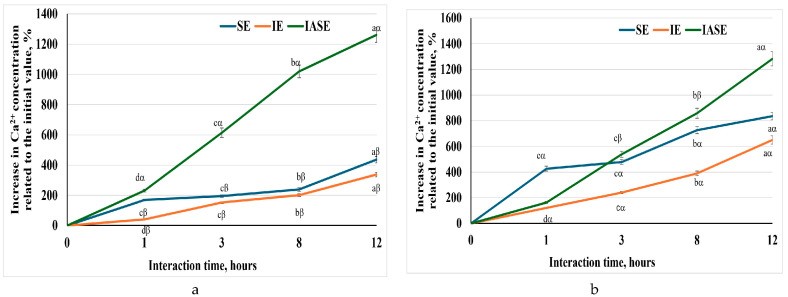
The increase in the Ca^2+^ ion concentration (%) related to the initial amount in wound exudate simulants during interaction with control dressing (**a**) and dressing containing 0.2% liposomal curcumin (**b**). SE is serous exudate, superficial wound; IE is inflammatory exudate, purulent exudate; IASE is infected exudate with abundant secretion. Values marked with different letters a, b, c, and d for the same simulant are significantly different. Values marked with different letters α and β for different simulants are significantly different (one-way ANOVA, Tukey’s test, *p* < 0.05).

The release of Ca^2+^ ions from dressings (Figure 8a,b) exhibits a pronounced dependence on both dressing composition and simulated wound environment. For the control dressing, Ca^2+^ release increased progressively with interaction time in all simulants, reflecting the gradual displacement of structural Ca^2+^ ions from the alginate “egg-box” network by monovalent ions present in the exudate. The highest release was observed in the infected exudate with abundant secretion (IASE), where the Ca^2+^ concentration increased from 231.58% at 1 h to 1261.40% at 12 h, indicating extensive matrix hydration and ion exchange. Intermediate values were recorded in the serous exudate (SE), where Ca^2+^ release increased from 169.33% to 436.00%, while the lowest release was observed in the inflammatory exudate (IE), increasing from 40.22% to 337.33%. The extensive release detected in IASE is likely associated with enhanced matrix hydration and structural destabilization promoted by the more acidic and biochemically complex environment, which facilitates ion exchange and progressive alginate network reorganization. The incorporation of 0.2% liposomal curcumin markedly altered the calcium-release behavior of the alginate matrix. In SE and IE, the D-0.2 dressing consistently released greater amounts of Ca^2+^ than the control throughout the experimental period.

In SE, Ca^2+^ release increased from 426.67% at 1 h to 836.00% at 12 h, while in IE the corresponding values increased from 121.33% to 649.33%. These findings suggest that the incorporation of liposomal curcumin modifies the internal architecture of the alginate network, reducing local cross-linking density and facilitating matrix relaxation during swelling. In contrast, the behavior in IASE differed substantially. Although Ca^2+^ release increased continuously from 163.16% at 1 h to 1282.46% at 12 h, the control dressing released greater amounts of calcium than D_0.2 during the first 8 h of incubation, indicating that the influence of liposomal curcumin on ion release is strongly dependent on the biochemical composition of the surrounding environment. Comparative analysis of the two formulations demonstrated that the presence of liposomal curcumin generally enhanced calcium liberation in low- and moderate-complexity exudates (SE and IE), whereas this effect was attenuated in the highly exuding infected environment. Nevertheless, both dressings converged toward similar calcium-release levels at prolonged incubation times, suggesting the establishment of an equilibrium state characterized by extensive alginate network restructuring. The Ca^2+^ release profiles showed a close relationship with the observed hemostatic response. In SE and IE, the greater calcium release exhibited by D_0.2 correlated with the pronounced reduction in prothrombin time observed after 3 h, supporting the role of calcium ions as essential cofactors (Factor IV) in prothrombinase complex formation and thrombin generation. In IASE, however, the faster coagulation profile of D_0.2 was not accompanied by higher calcium release, indicating that additional mechanisms beyond total calcium liberation contribute to hemostatic regulation under infected, highly exuding conditions. Such mechanisms may include localized ion distribution at the dressing–exudate interface, matrix–protein interactions, and microstructural modifications induced by liposomal curcumin. From a clinical perspective, these findings demonstrate that the D_0.2 dressing can generate a sustained procoagulant microenvironment while adapting its response to different wound conditions.

The combination of controlled calcium delivery and bioactive matrix remodeling may be particularly advantageous for the management of chronic and infected wounds, where rapid hemostasis and effective exudate handling are essential for preventing excessive blood loss and supporting tissue repair. The work of Goh et al. [42] reported that approximately 43% of the calcium was released from alginate-based films after 1 h of incubation in simulated wound fluid containing 42 mM Na^+^ and 2.5 mM Ca^2+^ at pH 4.0, whereas a release of 41% was observed under the same conditions at pH 8.0.

**Figure 9:**
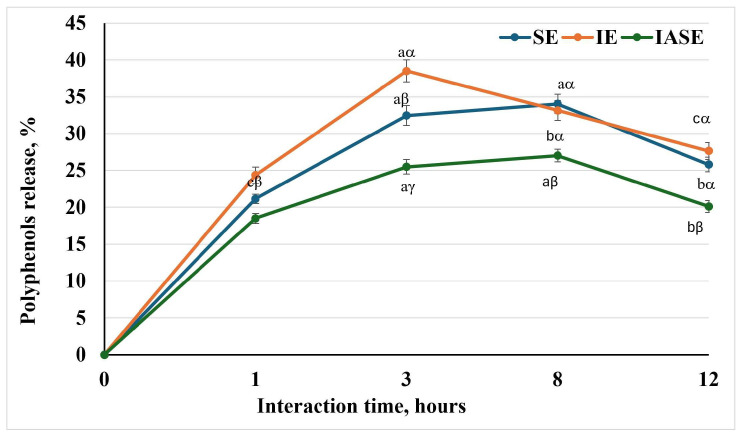
The increase in the polyphenols amount (%) related to the initial amount in wound exudate simulants during interaction with a dressing containing 0.2% liposomal curcumin. SE is serous exudate, superficial wound; IE is inflammatory exudate, purulent exudate; IASE is infected exudate with abundant secretion. Values marked with different letters a, b, c for the same simulant are significantly different. Values marked with different letters α, β and γ for the different simulants are significantly different (one-way ANOVA, Tukey’s test, *p* < 0.05).

The release profile ofpolyphenolic compounds (Figure 9) from D_0.2 exhibited distinct differences among the three wound exudate simulants, reflecting the influence of the surrounding biochemical environment on diffusion and matrix–medium interactions.

In all cases, an initial burst release was observed at 3 h, followed by a maximum at 8 h. A slight decrease was then noted at 12 h. This profile is consistent with diffusion-controlled release from liposomal structures. Secondary processes, such as protein binding, adsorption to the alginate network, and partial oxidation of curcumin, may contribute to the later-stage decrease. Among the tested exudate simulants, EI and SS generally promoted higher apparent polyphenol release compared to EIS, suggesting that the inflammatory and serous environments favor faster matrix hydration and enhanced diffusion. The IASE simulant likely induces stronger biomolecular interactions that reduce the fraction of freely detectable compounds. When interpreted alongside Ca^2+^ ions release, a clear hierarchical mechanism emerges. Calcium showed a strong, time-dependent increase in all simulants, with the most pronounced release observed in IASE/EIS conditions. This indicates that ion-exchange processes between the alginate matrix and the exudate are the dominant driver of the physicochemical behavior of the system. In contrast, polyphenol release appears to act as a transient modulatory factor, primarily influencing the early stages of interaction (0–3 h), when its availability may contribute to the initial prolongation of prothrombin time. As calcium availability increases over time, the hemostatic response becomes increasingly governed by Ca^2+^-mediated activation of the coagulation cascade, leading to the observed reduction and subsequent stabilization of PT values. Zamani et al. [28] reported the development of curcumin-loaded alginate hydrogels that provided sustained curcumin release for up to 3 days. The controlled release behavior was attributed to the alginate network, which modulated curcumin diffusion and enhanced its retention at the wound site. Furthermore, the hydrogels showed minimal blood-clotting potential, indicating good compatibility with blood-contacting tissues.

Overall, the integrated analysis of polyphenol release, Ca^2+^ ions dynamics, and PT results indicates a synergistic but hierarchically organized mechanism. Calcium ions released from the alginate matrix represent the primary determinant of coagulation kinetics, while polyphenolic compounds provide an early-stage, environment-dependent modulatory effect that is progressively outweighed by sustained Ca^2+^-driven procoagulant activity.

### 2.9. Fibrin Characterization

#### 2.9.1. Fibrin Appearance and Morphology

Table 1 illustrates the macroscopic appearance and structural morphology of the fibrin clots formed following a 3 h interaction between the dressings and the simulated wound exudates. The fibrin clot morphologies generated in the presence of D_0 dressings were strongly influenced by both dressing composition and the physicochemical characteristics of the wound exudate simulants. Calcium alginate is recognized as an effective hemostatic material due to its ability to release Ca^2+^ ions, which serve as essential cofactors for the assembly of coagulation enzyme complexes and subsequent thrombin generation [43,44]. Upon hydration, the calcium-alginate matrix undergoes ion exchange and swelling, facilitating the diffusion of Ca^2+^ into the surrounding medium and promoting fibrin formation. In the D_0 dressing, the fibrin network formed in serous exudate (SE) displayed a dense and highly interconnected three-dimensional architecture. This environment was characterized by physiological pH (7.0), low protein content, and the lowest calcium concentration (1 mM), conditions that favor regular fibrin protofibril elongation and branching. Previous studies have demonstrated that fibrin structure is highly dependent on local polymerization conditions and that controlled fibrin assembly typically produces continuous, interconnected fibrillar networks [43,44]. The radial and cohesive morphology observed in SE is therefore consistent with relatively unhindered fibrin polymerization occurring under near-physiological conditions. In contrast, the fibrin structures formed in inflammatory exudate (IE) appeared more compact and condensed. Compared with SE, the IE simulant contained higher concentrations of calcium (3 mM) and albumin (10 g/L), together with a slightly acidic pH (6.5). Albumin has been shown to influence fibrin assembly by modulating the lateral aggregation of fibrin polymers and altering fibril ultrastructure [45,46].

Consequently, the more condensed architecture observed in IE may reflect the combined effects of increased calcium availability, which can modify fibrin polymerization behavior, and enhanced macromolecular crowding arising from elevated protein concentration. Similar alterations in fibrin organization under varying physicochemical conditions have been described previously for plasma-derived fibrin networks [43,44]. The most pronounced structural disruption was observed in infected exudate (IASE), where the clot exhibited a fragmented and discontinuous architecture. This simulant contained the highest calcium concentration (5 mM), the greatest protein burden (20 g/L albumin plus hydrolyzed casein), elevated lysozyme levels, and the lowest pH (6.0). Fibrin assembly is known to be sensitive to environmental pH, ionic composition, and protein concentration, all of which can influence fibril growth, branching, and network organization [43,44]. Therefore, the fragmented morphology observed in IASE likely reflects the cumulative effects of an acidic environment and increased macromolecular crowding on fibrin assembly. While albumin and other soluble proteins may contribute to these structural changes, the present data do not permit attribution of the observed morphology to a single component.

The incorporation of 0.2% liposomal curcumin into the alginate matrix altered fibrin organization while preserving clot formation in all simulated wound environments. Curcumin possesses documented anticoagulant properties and has been shown to prolong prothrombin time (PT) and activated partial thromboplastin time (aPTT) while inhibiting both thrombin and factor Xa activity [31]. Consequently, the release of low concentrations of curcumin from the dressing may have moderated coagulation kinetics without completely suppressing fibrin generation. In the SE environment, D_0.2 produced thicker and more organized fibrillar assemblies than the corresponding D_0 formulation. This observation is consistent with established fibrin polymerization theory, which indicates that slower fibrin formation can promote enhanced lateral protofibril aggregation and the development of thicker fibrillar structures [43,44]. The presence of curcumin may therefore have subtly attenuated coagulation kinetics, allowing greater structural organization during network assembly. A similar trend was observed in IE, where D_0.2 generated dense and highly aligned fibrillar bundles rather than the more compact morphologies observed for D_0. Given the elevated protein concentration present in this environment, modulation of coagulation kinetics by curcumin may have facilitated more orderly fibrin assembly despite increased macromolecular crowding. This interpretation is consistent with the reported anticoagulant activity of curcumin and the known relationship between clotting kinetics and fibrin ultrastructure [31,44]. Under the challenging conditions of IASE, fibrin formation remained heterogeneous. However, D_0.2 produced localized fan-like fibrillar domains rather than the highly discontinuous structures observed for D_0. Although the precise mechanism cannot be established from morphological observations alone, these findings suggest that curcumin may partially modulate fibrin assembly under adverse physicochemical conditions. The resulting architecture indicates that fibrin polymerization remained locally organized despite the acidic and highly protein-rich environment. Nevertheless, this interpretation should be regarded as a hypothesis based on structural observations and would require further biochemical investigation to confirm the underlying mechanisms.

The morphological modulation of the fibrin network by dressings across diverse wound simulants carries profound clinical implications for thrombus stability and fibrinolytic susceptibility. Under serous conditions (SE), the transition from the dense, interconnected meshwork to the thicker, highly organized fibrillar assemblies represents a clinically favorable shift. Thicker fibers yield larger geometric pores, significantly enhancing permeability to plasminogen and tissue plasminogen activator (tPA), thereby optimizing clot breakdown and preventing hypertrophic scarring [47]. Conversely, in inflammatory environments (IEs), macromolecular crowding and elevated calcium typically drive a hyper-condensed, compact clotting phenotype in D_0 [45]. This hyper-condensed matrix represents a pathological architecture directly linked to an elevated risk of thromboembolism and is a classic biomarker in metabolic disorders like diabetes, where chronic low-grade inflammation alters fibrinogen modification [48]. Due to their ultra-fine pore structures, these clots exhibit extreme resistance to lysis, meaning they persist abnormally within the vasculature, fail to dissolve under therapeutic thrombolysis, and dramatically increase the clinical risk of ischemic stroke and deep vein thrombosis [49]. The incorporation of liposomal curcumin (D_0.2) mitigates this prothrombotic architecture, instead organizing the fibrin into dense, highly aligned bundles that offer directional mechanical stability and vital contact guidance for cellular infiltration. Most critically, in infected exudates (IASE), where severe physicochemical stress induces a fragmented and discontinuous matrix (D_0) that poses an imminent clinical risk of hemostatic failure, the formulation partially restores structural cohesion by producing localized, fan-like fibrillar domains. This local structural rescue maintains minimal mechanical integrity, safeguarding the clot against shear-induced fracturing and helping to compartmentalize the localized infection.

Overall, the results indicate that calcium alginate primarily functions as a procoagulant substrate through the local release of Ca^2+^ ions, whereas the incorporation of liposomal curcumin appears to influence the kinetics of fibrin assembly. The final clot architecture is therefore governed by a complex interplay between dressing composition and the biochemical properties of the surrounding wound environment, particularly calcium concentration, pH, and protein content. The more organized fibrillar structures observed for D_0.2 suggest that low concentrations of curcumin can modulate fibrin network formation without preventing clot generation, potentially improving clot organization across diverse wound microenvironments.

#### 2.9.2. Protein Secondary Structure in Fibrin

Fibrin, the principal structural component of the hemostatic clot, exhibits a secondary structure composed predominantly of α-helices, β-sheets, and turns/random coils [50]. Table 2 presents the proportions of protein secondary structures in fibrin networks formed during the PT assay under simulated wound exudate conditions after 12 h of dressing–wound simulant interaction. FTIR analysis of fibrin formed in the presence of the D_0 dressing showed that the simulated wound exudate significantly influenced the protein secondary structure of the clot. Distinct conformational profiles were observed in SE, IE, and IASE, indicating that the biochemical composition of the exudate affected fibrin organization and stability.

In SE, fibrin exhibited predominantly α-helix (28.85%) and intra-molecular β-sheet (36.66%) structures, suggesting a relatively ordered and compact protein conformation. The low proportion of intermolecular β-sheets (15.61%) and the absence of random coil (RC) structures indicate limited protein unfolding and aggregation. This profile is consistent with the formation of a stable fibrin network under the less aggressive biochemical conditions characteristic of serous wounds.

In IE, the fibrin structure showed higher 3/10 α-helix (12.43%) and intermolecular β-sheet (27.45%) contents than those observed in SE. These changes suggest enhanced protein–protein interactions and partial rearrangement of fibrin chains under inflammatory conditions. The appearance of β-turn structures (2.04%) further indicates increased conformational flexibility and structural adaptation in response to inflammatory mediators present in the exudate.

Nevertheless, the absence of random coil structures demonstrates that the fibrin network largely preserved its ordered conformation despite inflammatory stress. The most pronounced structural alterations were observed in IASE. Fibrin formed under these pathological conditions exhibited markedly lower α-helix (20.53%) and intra-molecular β-sheet (4.79%) contents. These changes were accompanied by substantial increases in random coil structures (15.47%) and side-chain contributions (31.83%). Together, these findings indicate extensive protein unfolding, disruption of ordered secondary structures, and increased exposure of amino acid side chains. The elevated intermolecular β-sheet content (22.19%) may also reflect enhanced protein aggregation or abnormal fibrin assembly induced by the infected exudate. Such conformational destabilization could compromise both the mechanical integrity and functional performance of the fibrin clot.

FTIR analysis further demonstrated that bioactive compounds released from the D_0.2 dressing influenced fibrin secondary structure organization. In SE, fibrin contained 25.31% α-helices together with a relatively high proportion of intra-molecular β-sheets (27.60%). Increased 3/10 α-helix content (16.58%) and the presence of β-turns (2.43%) suggest enhanced conformational flexibility and controlled protein rearrangement during clot formation. The moderate proportion of intermolecular β-sheets (18.99%) indicates limited protein aggregation, whereas the absence of random coil structures reflects preservation of an ordered fibrin architecture. Overall, these findings indicate that the D_0.2 dressing-maintained fibrin structural integrity under SE conditions.

Under IE conditions, fibrin exhibited a lower α-helix content (16.92%) while maintaining a substantial proportion of intra-molecular β-sheet structures (26.49%). The appearance of random coils (7.76%) together with increased β-turn content (4.96%) indicates partial protein unfolding and greater molecular flexibility within the inflammatory environment. Despite these changes, the relatively balanced distribution of ordered and disordered structures suggests that the fibrin network retained its overall structural organization. The increased side-chain contribution (18.68%) may reflect greater exposure of amino acid residues caused by conformational rearrangements.

The most pronounced protective effect of D_0.2 was observed under IASE conditions. Fibrin exhibited the highest intra-molecular β-sheet content (37.39%) among all experimental groups, together with a reduced intermolecular β-sheet proportion (10.20%). This structural profile suggests a more compact and internally stabilized fibrin network with a lower tendency toward protein aggregation. Although β-turns (5.23%) and random coils (4.96%) were present, their proportions remained substantially lower than those measured for the control dressing under the same conditions. Likewise, the reduced side-chain contribution (17.00%) indicates less protein unfolding and decreased exposure of hydrophobic amino acid residues. These findings suggest that liposomal curcumin protects fibrin against structural destabilization under infected wound conditions.

Comparison of the D_) and D_0.2 dressings revealed marked differences in fibrin molecular organization. In SE, D_0.2 reduced the proportions of α-helices and intra-molecular β-sheets while increasing 3/10 α-helices, β-turns, and intermolecular β-sheets. These changes indicate controlled structural rearrangement without significant destabilization. Under IE conditions, D_0.2 promoted higher intra-molecular β-sheet and β-turn contents while inducing only moderate random coil formation, suggesting adaptive conformational flexibility during inflammation.

The greatest differences between the two dressings were observed in IASE. Compared with the control, D_0.2 markedly reduced random coil structures (4.96% vs. 15.47%) and side-chain exposure (17.00% vs. 31.83%). At the same time, it substantially increased intra-molecular β-sheet content (37.39% vs. 4.79%). These structural changes indicate that liposomal curcumin limited protein unfolding and promoted a more ordered fibrin conformation under the highly aggressive conditions of infected wound exudate. Furthermore, the lower intermolecular β-sheet content suggests reduced abnormal protein aggregation.

Overall, these findings indicate that incorporation of 0.2% liposomal curcumin into the alginate dressing modulated fibrin secondary structure and promoted preservation of ordered protein conformations, particularly under inflammatory and infected conditions. Quantitative FTIR analysis also supports the macroscopic observations of fibrin morphology, demonstrating that D_0.2 acts as a molecular stabilizer under microenvironmental stress.

In SE, the transition from the relatively rigid alginate network to a flexible three-dimensional fibrin architecture was associated with high retention of native α-helices (25.31%) together with flexible β-turns (2.43%), which may prevent premature collapse of fibrin chains. Under IE conditions, the formation of dense, vertically aligned fibrillar structures corresponded to a highly ordered intra-molecular β-sheet framework (26.49%). At the same time, a moderate increase in random coils (7.76%) may provide the structural flexibility required for protofibril alignment during macromolecular crowding.

The most pronounced structural differences were observed in IASE. In the D_0 dressing, extensive protein unfolding was reflected by the marked reduction in intra-molecular β-sheets (4.79%) together with substantial increases in random coils (15.47%) and exposed side chains (31.83%). In contrast, fibrin formed in the presence of D_0.2 maintained a considerably more ordered structure. Random coil content decreased to 4.96%, side-chain exposure was reduced to 17.00%, and intra-molecular β-sheet content increased to 37.39%. These molecular features closely correspond to the observed macroscopic morphology of resilient, fan-like fibrin bundles organized into discrete structural domains. Together, these observations suggest that liposomal curcumin stabilizes compact and ordered fibrin conformations capable of maintaining hemostatic integrity under severe infectious stress.

The improved fibrin organization may be attributed, at least in part, to interactions between curcumin and fibrinogen during clot formation. Curcumin has been reported to bind strongly to fibrinogen through hydrophobic interactions, with an association constant of approximately 10^5^ M^−1^ [51,52]. This interaction may modify the local molecular environment of fibrinogen and influence its conversion into fibrin, thereby affecting fibrin assembly and network architecture. In addition, fibrinogen-bound curcumin is protected from hydrolytic degradation, prolonging its biological activity at the wound site. The sustained presence of curcumin, together with its well-established antioxidant and anti-inflammatory properties, may reduce oxidative and inflammatory damage to fibrin fibers and promote the formation of a more ordered and mechanically stable fibrin network. These effects are likely to be particularly important in inflammatory and infected wound conditions, where excessive proteolytic activity and oxidative stress compromise clot integrity and delay healing.

The results indicate that the D_0.2 dressing promoted the formation of a more organized and structurally stable fibrin network, particularly under inflammatory and infected exudate conditions. Clinically, these findings are highly relevant because chronic and infected wounds are characterized by excessive proteolytic activity, oxidative stress, persistent inflammation, and impaired coagulation, all of which destabilize fibrin clots and delay healing. The reduction in random coil structures and side-chain exposure observed in IASE indicates lower protein unfolding and denaturation, suggesting improved preservation of fibrin integrity. Consequently, the clot may exhibit greater mechanical stability, increased resistance to premature fibrinolysis, improved retention of platelets and growth factors, and more effective temporary wound sealing. Furthermore, the increase in intra-molecular β-sheet structures together with the reduction in intermolecular β-sheet aggregation suggests formation of a more compact and functional fibrin network. Such structural organization may support faster hemostasis, provide a more effective scaffold for cell migration, promote tissue regeneration, and potentially reduce bleeding complications in highly exuding or infected wounds.

Taken together, the experimental findings indicate that the performance of the D_0.2 dressing arises from the synergistic interaction between the calcium-crosslinked alginate matrix and liposomally encapsulated curcumin. The selected curcumin concentration preserved the structural integrity and flexibility of the dressing while modifying the hydrated polymer network sufficiently to enhance fluid uptake, dimensional responsiveness, and water vapor transmission. At the same time, liposomal encapsulation enabled controlled curcumin release without compromising the calcium-mediated functionality of the alginate matrix. This balance generated the characteristic biphasic hemostatic response, in which the initial effects of curcumin release were progressively replaced by calcium-driven coagulation as ion exchange proceeded during hydration. The improved preservation of fibrin secondary structure under inflammatory and infected conditions further indicates that the released curcumin contributes not only to the modulation of coagulation kinetics but also to the stabilization of the developing fibrin network. The optical properties of the dressing provide an additional functional advantage by attenuating ultraviolet–blue radiation while maintaining favorable light interaction in the red and near-infrared spectral regions. Collectively, these results demonstrate that the beneficial performance of the D_0.2 formulation cannot be attributed to a single property of curcumin or alginate alone but rather to the coordinated interplay between matrix architecture, controlled bioactive release, calcium ion availability, and fibrin stabilization.

Several curcumin-loaded hydrogel dressings based on alginate or alginate-containing polymeric systems have demonstrated high swelling capacity, sustained curcumin release, and enhanced wound-healing performance through improved tissue regeneration, antibacterial activity, and cytocompatibility [24,25,26,27,28]. For example, bilayer alginate/chitosan hydrogels incorporating curcumin–β-cyclodextrin inclusion complexes exhibited favorable swelling behavior, appropriate water vapor transmission, and sustained drug release, while injectable thermoresponsive alginate hydrogels and gelatin/alginate semi-interpenetrating polymer network hydrogels promoted prolonged curcumin delivery and accelerated wound closure in vivo [24]. Similarly, alginate/gelatin composite sponges containing curcumin-loaded electrospun fibers demonstrated rapid hemostatic activity associated with effective blood absorption and controlled release [27]. The present study demonstrated comparable swelling behavior and controlled curcumin release while providing additional mechanistic insight into the interactions between the dressing and the wound microenvironment. Unlike previous reports, the hemostatic performance was systematically evaluated under three simulated wound exudate environments representing serous, inflammatory, and infected wounds. Moreover, the coordinated release of curcumin and calcium ions generated a biphasic coagulation response, revealing the dynamic interplay between transient anticoagulant effects and calcium-mediated clot formation. Quantitative FTIR analysis further demonstrated that the incorporation of liposomal curcumin preserved fibrin secondary structure by reducing protein unfolding and promoting intra-molecular β-sheet organization, particularly under inflammatory and infected conditions. This molecular-level evaluation extends beyond the conventional characterization of swelling, drug release, and biological performance commonly reported for curcumin-based wound dressings, providing a more comprehensive understanding of how the dressing modulates both coagulation kinetics and fibrin organization during the early stages of wound healing.

## 3. Conclusions

An alginate-based wound dressing incorporating 0.2% liposomal curcumin was successfully developed and demonstrated favorable physicochemical and hemostatic properties. Among the formulations containing 0–0.5% liposomal curcumin, the 0.2% formulation exhibited the most favorable macroscopic appearance and mechanical flexibility while maintaining the intended bioactive loading. In contrast, higher liposomal curcumin concentrations increased brittleness and promoted visible phase separation. The incorporation of 0.2% liposomal curcumin enhanced the physicochemical performance of the alginate dressing. After 12 h, the areal swelling ratio reached 172.04% in serous exudate compared with 140.51% for the control dressing, while maintaining the characteristic hydration behavior of the alginate matrix and exhibiting a higher water vapor transmission rate during prolonged hydration. Controlled-release studies demonstrated early curcumin release, followed by sustained Ca^2+^ liberation, resulting in a biphasic hemostatic response. After 3 h, the D_0.2 dressing reduced the PT increase to 3.50% in serous exudate, 8.06% in inflammatory exudate, and 13.89% in infected exudate, indicating faster coagulation than the control dressing. FTIR analysis further demonstrated improved preservation of fibrin secondary structure, particularly under infected wound conditions, where intra-molecular β-sheet content increased from 4.79% to 37.39%, while random coil structures decreased from 15.47% to 4.96%. These changes indicate reduced protein unfolding, enhanced fibrin structural stability, and the formation of a more organized fibrin network. Overall, the developed 0.2% liposomal curcumin–alginate dressing combines effective exudate management, adaptive hemostatic regulation, and stabilization of the fibrin matrix. These findings highlight its potential as a multifunctional wound dressing for the management of chronic, highly exuding, and infected wounds.

The main limitations of this study include the absence of quantitative mechanical characterization, which prevents definitive assessment of the dressing’s mechanical robustness, handling characteristics, and durability during clinical application. Consequently, although the dressing exhibited favorable physicochemical properties, its mechanical suitability for wound management remains to be experimentally verified. In addition, the lack of cytocompatibility, antimicrobial, and in vivo wound-healing studies limits the translation of the present in vitro findings into biological performance and clinical efficacy. Therefore, the observed improvements in swelling behavior, hemostatic response, and fibrin organization should be interpreted as evidence of functional potential rather than definitive proof of therapeutic effectiveness. Furthermore, the hemostatic evaluation relied primarily on prothrombin time measurements, providing only a partial assessment of coagulation. The proposed mechanisms involving curcumin release, calcium ion exchange, and fibrin stabilization were inferred from indirect observations rather than demonstrated by dedicated mechanistic experiments. Accordingly, these mechanistic interpretations should be regarded as plausible hypotheses that require further experimental validation.

Future research should include a comprehensive mechanical characterization of the dressing, including tensile strength, elasticity, and durability, to confirm its suitability for clinical use. Additional studies should evaluate cytocompatibility, cell proliferation, and antimicrobial activity to validate the biological safety and therapeutic potential of the formulation. A broader assessment of hemostatic performance, including platelet activation and coagulation pathway analyses, would further clarify the mechanisms underlying the observed effects. Finally, in vivo wound-healing studies are essential to confirm the dressing’s efficacy, biocompatibility, and capacity to promote tissue repair under clinically relevant conditions.

## 4. Materials and Methods

### 4.1. Materials

Sodium alginate (viscosity of 25.7 cps at 25 °C, mannuronic/guluronic ratio of 35/65—data supplied by the manufacturer, Merck (Rahway, NJ, USA)), glycerol, 2,2-diphenyl-1-picrylhydrazyl (DPPH), Folin–Ciocalteu reagent, Na_2_CO_3_, NaCl, CaCl_2_, KCl, MgCl_2_, phosphate buffer, bovine albumin, glucose, lysozyme, urea, elastase, lactic acid, L-cysteine, and hydrolyzed casein were purchased from Merck (Rahway, NJ, USA). Liposomal curcumin was provided by Remedia Laboratories (Bucharest, Romania). Assay kits for the measurement of prothrombin time (PT), and plasma (Control Level 1) were purchased from BioSystems (Barcelona, Spain).

### 4.2. Preparation of Alginate-Based Dressings

Five formulations were prepared as described in Table 3. Sodium alginate was mixed in distilled water under continuous stirring until a homogeneous solution was obtained. Glycerol was added as a plasticizer, and the mixture was stirred further until fully incorporated. Finally, liposomal curcumin was added and uniformly dispersed in the solution under continuous stirring. Prepared solutions were cast into Petri dishes (9 cm in diameter) to form thin films. The Petri dishes with films were dried in an oven at 35 °C until complete solvent evaporation and formation of dressings. After drying for 24 h at 30 °C, the dressings were immersed for 15 min in a 500 mL solution of CaCl_2_ 2%. Following crosslinking, the dressings were removed and rinsed gently with distilled water to remove excess calcium ions. The samples were then dried at 30 °C for 24 h to obtain Ca^2+^-crosslinked alginate dressings. The samples were then dried at 30 °C for 24 h. Upon exposure to wound exudate, these crosslinked dressings rehydrate, forming a hydrated three-dimensional alginate network capable of absorbing exudate and releasing the incorporated liposomal curcumin.

### 4.3. Macroscopic Appearance

Photographic images of the dressing formulations were obtained using a Canon 80D digital single-lens reflex (DSLR) camera. The samples were visually examined for uniformity, structural integrity, and surface homogeneity, along with the distribution and intensity of color resulting from liposomal curcumin incorporation. Special attention was paid to signs of phase separation, aggregation, or surface irregularities that might indicate instability or uneven dispersion of the liposomal system. A comparative visual analysis was also conducted to assess variations in color intensity and overall appearance depending on liposomal curcumin concentration. The formulation that exhibited the best overall performance in terms of these parameters was selected for further analysis, together with the alginate-only dressing, which served as the control sample.

### 4.4. Characterization of Selected Dressings

#### 4.4.1. Morphological Assessment

The selected dressings underwent morphological assessment based on images captured via BRESSER Biolux Touch microscope (BRESSER GmbH, Rhede, Germany).

#### 4.4.2. Swelling Behavior of Selected Dressings

The swelling behavior of hydrogel dressings is a critical parameter from a medical perspective, as it governs their ability to absorb and retain wound exudate while maintaining a moist environment conducive to healing. Appropriate swelling ensures effective fluid management, preventing both desiccation and maceration of surrounding tissues. Additionally, swelling influences the mechanical stability and conformability of the dressing, enabling intimate contact with the wound bed. It also regulates the diffusion of oxygen, nutrients, and therapeutic agents, thereby impacting tissue regeneration and infection control. Therefore, a balanced swelling profile is essential for optimal wound care performance.

The fluid absorption capacity and swelling kinetics of the selected dressings were quantitatively evaluated by determining their equilibrium swelling ratio. Briefly, pre-weighed dry dressing samples (1 cm × 1 cm) were fully immersed in 25 mL of three different simulated wound exudates (compositions provided in Table 4) and incubated at 37 °C for 24 h to ensure maximum fluid uptake.

The compositions of the wound exudate simulants were developed by integrating standardized artificial wound fluid formulations [53] with the reported physiological and biochemical characteristics of human wound exudate at different stages of healing and infection. The electrolyte base, consisting of NaCl, KCl, CaCl_2_, MgCl_2_, and phosphate buffer, was included to reproduce the ionic strength and extracellular fluid origin of wound exudate. Wound exudate is primarily derived from plasma ultrafiltrate and therefore has an electrolyte composition similar to that of serum. NaCl serves as the principal osmotic component, whereas the divalent ions Ca^2+^ and Mg^2+^ support coagulation, enzymatic activity, and cell-signaling processes involved in tissue repair [54]. The protein content, represented by bovine serum albumin and hydrolyzed casein, was adjusted to reproduce the well-documented increase in protein concentration from serous to infected wounds. Bovine serum albumin was selected as a surrogate for plasma-derived proteins because it is widely used in simulated wound fluid models [55]. Hydrolyzed casein represents the proteolytic degradation products commonly found in chronic and infected wound environments. Metabolic constituents, including glucose, lactate, and urea, were incorporated to mimic clinically observed changes in wound biochemistry.

These changes include glucose depletion caused by microbial consumption, elevated lactate levels associated with hypoxia and inflammation, and increased urea concentrations resulting from tissue degradation and systemic diffusion [56]. Lysozyme and L-cysteine were included to represent innate immune activity and changes in the redox and inflammatory microenvironment that characterize progressively more severe wounds [55]. Finally, the pH of each simulant was adjusted to reproduce the transition from near-neutral conditions in superficial wounds to more acidic conditions in infected and highly inflamed wounds. This approach generated a graded model that captures the key physicochemical changes reported in the wound-fluid literature.

At predefined time intervals (1, 2, 3, and 12 h), the samples were carefully removed from the exudates. Excess surface exudate was gently blotted using filter paper to remove unbound liquid, and the wet weight was immediately measured using an analytical balance. The swelling capacity was then calculated gravimetrically according to Equation (1):

Swelling capacity (%) = (Weight of swollen dressing − Weight of dry dressing) ∗ 100/Weight of dry dressing
(1)

#### 4.4.3. Dimensional Stability of Selected Dressings

Quantifying the dimensional stability of wound dressings provides essential information on their macroscopic morphomechanical behavior, structural integrity, and potential clinical performance. Because alginate networks are inherently anisotropic, monitoring total surface area expansion together with longitudinal and transverse dimensional changes prevents localized directional distortions from being overlooked. From a clinical perspective, evaluating two-dimensional geometric stability is important for ensuring optimal anatomical conformity and sustained coverage of the wound bed. Good dimensional stability enables the dressing to absorb wound exudate without excessive shrinkage or expansion. Shrinkage may expose the wound bed to external contaminants, whereas excessive expansion can increase the risk of periwound maceration. Furthermore, changes in surface area are directly associated with relaxation of the polymer network and expansion of its internal pore structure. Consequently, bidimensional stability provides a useful predictor of both fluid uptake dynamics and the diffusion-controlled release of curcumin-loaded liposomes into the wound microenvironment.

To assess dimensional stability, dressing samples (1 cm × 1 cm) were immersed in 25 mL of three distinct simulated wound exudates (compositions listed in Table 4) and maintained at 37 °C for 24 h to achieve equilibrium swelling. At specified time points (1, 2, 3, and 12 h), the samples were withdrawn from the solutions. Surface-adhered exudate was carefully removed by blotting with filter paper, after which the length and width of each sample were promptly measured using a digital caliper. The areal swelling ratio was calculated using Equations (2)–(4):

Initial surface area (A_0_) = Length_0_ ∗ Width_0_ (2)

Swollen surface area (A_S_) = Length_S_ ∗ Width_S_ (3)

Dimension stability (%) = (A_S_ − A_0_) ∗ 100/A_0_ (4)

where Length_0_, Length_S_ are the exact length of the dry dressing measured right before it is placed into the swelling exudates simulants, and at a specific time point, respectively; Width_0_, Width_S_ are the exact width of the dry dressing measured right before immersion, and at a specific time point, respectively.

#### 4.4.4. Water Vapors Transmission Rate

The water vapor transmission rate (WVTR) was determined according to the method described by Sobral et al. [57], with minor adaptations for the dressing samples. Dressing samples without visible defects were fixed over the opening of permeation cells (effective permeation area of 1 mm^2^) containing approximately 3 g of silica gel. An air gap of about 0.5 cm was maintained between the dressing surface and the silica gel to ensure unobstructed vapor diffusion. The assembled cells were placed in a desiccator containing distilled water and stored at (37 ± 0.5) °C to maintain a constant humidity gradient. The systems were weighed periodically to the nearest 0.0001 g, and during the final stage of the experiment, measurements were taken periodically until steady-state conditions were achieved. A plot of mass gain versus time was used to identify the steady-state region, and WVTR was subsequently calculated using the corresponding equation:

WVP (g·s^−1^·m^−1^·Pa^−1^) = w·x/(t·A·ΔP) (5)

where WVTR is water vapor transmission rate (g·s^−1^·m^−1^·Pa^−1^); w is the weight gain at steady-state, g; x is dressing sample thickness, m; t is the time of gain at steady state, s; A is the permeation area, m^2^ and ΔP is the difference in partial vapor pressure of the atmosphere with silica gel and distilled water (2546 Pa at 37 °C). The results are presented as the percentage increase in WVTR relative to the initial value.

#### 4.4.5. Reflectance Spectra and Chromatic Analysis of Selected Dressings

Reflectance spectral analysis was applied to the dressing containing liposomal curcumin to characterize its color-related compounds, while the alginate-only dressing, being white, was not subjected to spectral evaluation. Spectral data for the liposomal curcumin-enriched sample were collected over the 360–780 nm wavelength range, with ten replicate scans recorded at 1 nm intervals. All measurements were carried out using a YL4560 non-contact bench spectrophotometer (Shenzhen ThreeNH Technology Co., Ltd., Shenzhen, China).

The chromatic characteristics of the reflected light were evaluated only for the dressing containing liposomal curcumin, as the alginate-only sample exhibited a uniform white appearance. Average reflectance values were calculated across seven defined wavelength intervals of the visible spectrum: 380–449 nm (purple), 450–484 nm (blue), 485–499 nm (cyan), 500–564 nm (green), 565–589 nm (yellow), 590–624 nm (orange), and 625–760 nm (red). These spectral regions correspond to color-contributing compounds present in the liposomal curcumin-enriched dressing and provide insight into their optical behavior and distribution.

#### 4.4.6. Release Kinetics of Bioactive Compounds

The release kinetics of bioactive compounds from the selected dressings were evaluated by monitoring the increase in the concentrations of Ca^2+^ ions and polyphenols amount in wound exudate simulants. An amount of 0.5 g of dressing was immersed in 20 mL of wound exudate simulants, and samples of exudates were collected periodically.

Calcium concentration was measured using the Calcium Arsenazo III assay [58]. Briefly, calcium ions react with Arsenazo III (2,7-bis(2-arsonophenylazo)-1,8-dihydroxynaphthalene-3,6-disulfonic acid) at neutral pH to form a blue-colored complex, whose absorbance was measured at 650 nm using a BTS-350 Spectrophotometer (BioSystems, Barcelona, Spain). The results are presented as the percentage increase in Ca^2+^ concentration relative to the initial value.

Total polyphenol content was determined according to the method described by Wu et al. [59], adapted to our study. Briefly, 1 mL of wound exudate simulant was mixed with 5 mL of 10% (*v*/*v*) Folin–Ciocalteu reagent and 4 mL of 7.5% (*w*/*v*) Na_2_CO_3_ solution. The reaction mixture was incubated in the dark at 25 °C for 1 h, after which the absorbance was measured at 765 nm (Lambda 35 Spectrophotometer, PerkinElmer, Shelton, CT, USA). Total polyphenol content was quantified using a gallic acid calibration curve and expressed as milligrams of gallic acid equivalents (mg GAE) per gram of dry sample (Figure S1). The results are presented as the polyphenols release (%) relative to the initial amount. Since alginate does not contain polyphenolic compounds, the analysis was performed only on the alginate–liposomal curcumin dressing.

#### 4.4.7. In Vitro Hemostatic Evaluation of Wound Dressings

The hemostatic performance of wound dressings is strongly influenced by their physicochemical properties, which can modulate platelet activation, coagulation pathways, and overall clotting efficiency. The hemostatic effectiveness of the selected dressings was evaluated by measuring their ability to promote blood clotting under controlled in vitro conditions. Specifically, 0.5 g of each dressing was incubated at 37 °C in 20 mL of a wound exudate simulant. At predetermined time points (0, 1, 3, and 12 h), 1 mL aliquots of the simulant were collected and immediately analyzed. Coagulation activity was assessed using the prothrombin time (PT) assay, as it evaluates the extrinsic and common coagulation pathways primarily involved in tissue injury and acute bleeding, providing a physiologically relevant measure of the material-induced hemostatic response. The prothrombin time assay measures the time required for fibrin clot formation following the addition of calcium thromboplastin to plasma samples [58]. For hemostasis evaluation, three types of plasma samples were prepared for each assay: (i) lyophilized human plasma reconstituted according to the manufacturer’s instructions, serving as the positive control [58]; (ii) plasma mixed with wound exudate simulant (4:1 v:v), serving as the negative control; and (iii) plasma mixed with wound exudate simulant in which the dressing had been previously incubated (4:1 v:v), representing the test sample. All assays were performed at least in triplicate to ensure reproducibility. Results are expressed as a percentage increase over the negative control ± standard deviation.

#### 4.4.8. Fibrin Characterization

Wound healing and hemostasis are primarily initiated through tissue factor–mediated activation of the extrinsic coagulation pathway. Therefore, fibrin generated in the prothrombin time (PT) assay after 12 h of dressing–wound simulant interaction was considered the most physiologically relevant model for evaluating the effects of the dressing on fibrin morphology and structure.

**Figure 10:**
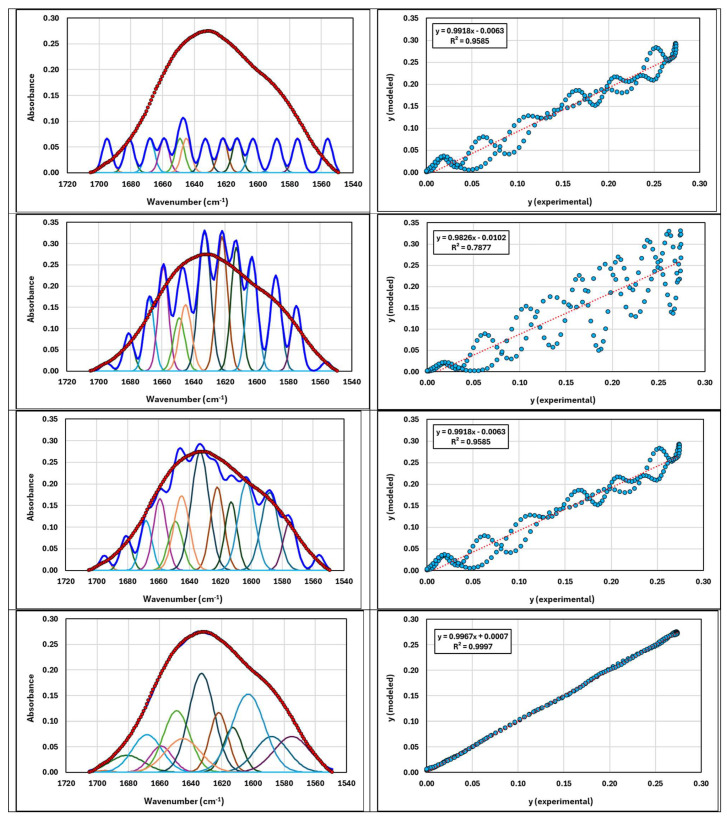
Sequences illustrating the iterative spectral deconvolution process used to analyze the secondary structure of fibrin. The red line represents the experimental spectrum. The thinner colored lines represent the individual distributions used for deconvolution. The blue line represents their sum.

FTIR spectra of fibrin clots generated during the PT assay were recorded using a PerkinElmer BX2 spectrometer (USA) equipped with a Pike Miracle ATR diamond crystal. Spectra were acquired over the range of 600–4000 cm^−1^ at a spectral resolution of 4 cm^−1^, with 50 scans accumulated for each sample. Prior to analysis, all spectra were normalized. The secondary structural composition of the fibrin networks was evaluated from the amide I region. Quantitative analysis was performed by Gaussian curve fitting (deconvolution) using band positions identified by second-derivative spectral analysis. The relative contributions of the following secondary structural elements were determined: high-frequency intramolecular β-sheet (β-sheet intra-HF) at 1700–1690 cm^−1^, β-turns at 1689–1670 cm^−1^, 3_10_-helix at 1669–1660 cm^−1^, α-helix at 1659–1649 cm^−1^, random coil at 1648–1640 cm^−1^, intramolecular β-sheet (β-sheet intra-M) at 1639–1626 cm^−1^, intermolecular β-sheet at 1625–1610 cm^−1^, and side-chain vibrations at 1609–1595 cm^−1^, according to the assignments reported by Zhao et al. [60]. The iterative spectral deconvolution procedures employed for the determination of secondary structure components are illustrated in Figure 10. Photographic images of the fibrin network were acquired using a Canon EOS 80D digital single-lens reflex (DSLR) camera (Canon Inc., Tokyo, Japan). The morphology of the fibrin samples was assessed using images captured with a BRESSER Biolux Touch microscope (BRESSER GmbH, Rhede, Germany).

### 4.5. Statistical Analysis

All experiments were carried out in triplicate, and the data are reported as mean values ± standard deviation. Statistical analysis was performed using one-way analysis of variance (ANOVA), followed by Tukey’s post hoc test to identify significant differences between groups, with a significance threshold set at *p * < 0.05.

## Figures and Tables

**Table 1:** Appearance and microscopic morphology of fibrin networks formed in prothrombin time assay after 12 h of dressings–simulated wound exudates interaction.

Wound Exudate Simulants	Control Dressing, D_0		Dressing with 0.2% Liposomal Curcumin D_0.2	
	Appearance	Microscopic Morphology	Appearance	Microscopic Morphology
Serous exudate (SE)	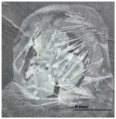	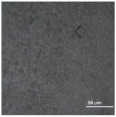	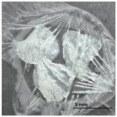	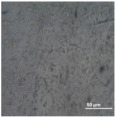
	Integrated, radial fibrous matrix, Distinct and thick radial strands; Continuous web anchoring;		Isotropic and interconnected radial web; Uniformly distributed porosity.	
Inflammatory exudate (IE)	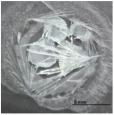	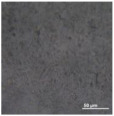	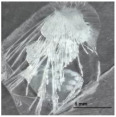	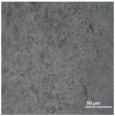
	Rigid and geometric crystalline plates; Flat, overlapping rigid sheets; Stiff, fractured intersections; Brittle.		Condensed and vertical streams; Thickest, heavily aggregated parallel cords; Locally dense core with large structural voids.	
Infected exudate with abundant secretion (IASE)	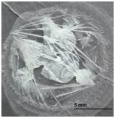	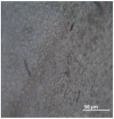	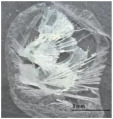	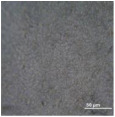
	Collapsing, fragmented planar shards; Partially degraded and brittle fragments; Isolated islands with massive gaps; Severely collapsing.		Fragmented, multi-centric independent domains; Feathered and fan-like radiating fiber sheets; Discontinuous network with gaps.	

**Table 2:** Proportions of protein secondary structures in fibrin networks formed during the prothrombin time assay after 12 h of dressing–simulated wound exudates interaction.

Simulated Wound Exudate	3/10 α-Helix (%)	α-Helix (%)	β-Sheet (Intra-hf) (%)	β-Sheet (Intra-m) (%)	β-Sheet (Inter) (%)	β-Turn (%)	Random Coil (%)	Side Chain (%)
Control dressing, D_0								
Serous Exudate (SE)	8.37 ± 0.30 ^b^	28.85 ± 1.03 ^a^	0.18 ± 0.01 ^b^	36.66 ± 0.89 ^a^	15.61 ± 0.49 ^b^	0.00 ± 0.00 ^b^	0.00 ± 0.00 ^a^	10.34 ± 0.30 ^a^
Inflammatory exudate (IE)	12.43 ± 0.38 ^a^	31.96 ± 0.97 ^a^	0.37 ± 0.01 ^b^	16.52 ± 0.33 ^a^	27.45 ± 1.14 ^a^	2.04 ± 0.05 ^b^	0.00 ± 0.00 ^b^	9.23 ± 0.37 ^b^
Infected exudate with abundant secretion (IASE)	4.12 ± 0.17 ^b^	20.53 ± 0.86 ^a^	0.15 ± 0.01 ^b^	4.79 ± 0.15 ^b^	22.19 ± 0.64 ^a^	0.93 ± 0.03 ^b^	15.47 ± 0.43 ^a^	31.83 ± 1.06 ^a^
Dressing containing 0.2% liposomal curcumin, D_0.2								
Serous Exudate (SE)	16.58 ± 0.51 ^a^	25.31 ± 0.68 ^b^	0.26 ± 0.01 ^a^	27.60 ± 0.86 ^b^	18.99 ± 0.53 ^a^	2.43 ± 0.03 ^a^	0.00 ± 0.00 ^a^	8.82 ± 0.27 ^b^
Inflammatory exudate (IE)	8.12 ± 0.22 ^b^	16.92 ± 0.47 ^b^	0.41 ± 0.01 ^a^	26.49 ± 1.08 ^b^	16.66 ± 0.34 ^b^	4.96 ± 0.10 ^a^	7.76 ± 0.20 ^a^	18.68 ± 0.67 ^a^
Infected exudate with abundant secretion (IASE)	5.14 ± 0.11 ^a^	19.36 ± 0.67 ^a^	0.72 ± 0.02 ^a^	37.39 ± 1.58 ^a^	10.20 ± 0.15 ^b^	5.23 ± 0.16 ^a^	4.96 ± 0.15 ^b^	17.00 ± 0.52 ^b^

Values marked with different letters (a, b) for the same protein secondary-structure component within the same simulated wound exudate are significantly different (one-way ANOVA, Tukey’s test, *p* < 0.05).

**Table 3:** Formulations of alginate-based dressings.

Ingredient	D_0	D_0.1	D_0.2	D_0.3	D_0.4	D_0.5
Alginate powder, g	1.86	1.86	1.86	1.86	1.86	1.86
Glycerin, mL	4.6	4.6	4.6	4.6	4.6	4.6
Liposomal curcumin	-	0.096	0.193	0.29	0.386	0.483
Distilled water, mL	88.6	88.6	88.6	88.6	88.6	88.6

**Table 4:** Composition of wound exudate simulants.

Component	Concentration of Component		
	SE (Serous Exudate, Superficial Wound)	IE (Inflammatory Exudate, Purulent Exudate)	IASE (Infected Exudate with Abundant Secretion)
NaCl	9 g/L	9 g/L	9 g/L
CaCl_2_	1 mM	3 mM	5 mM
KCl	4 mM	6 mM	8 mM
MgCl_2_	0.5 mM	1 mM	1.5 mM
Phosphate buffer	10 mM	10 mM	10 mM
Bovine albumin	5 g/L	10 g/L	20 g/L
Glucose	2 g/L	1.5 g/L	3 g/L
Lyzozime	0.02 g/L	0.05 g/L	0.1 g/L
Urea	2 mM	5 mM	10 mM
Lactic acid	1 mM	5 mM	15 mM
L-cysteine	0	0.05 g/L	0.2 g/L
Hydrolyzed casein	0	0.5 g/L	1 g/L
Elastase	0.01 U/mL	0.1 U/mL	0.5 U/mL
pH	7	6.5	6

## Data Availability

The original contributions presented in this study are included in the article/supplementary material. Further inquiries can be directed to the corresponding author.

## Supplementary Materials

The following supporting information can be downloaded at https://www.mdpi.com/article/10.3390/gels12070626/s1. Table S1: Chromatic components of alginate-based dressing contain 0.2% liposomal turmeric; Figure S1: Calibration curve for polyphenols release from dressings.

## Abbreviations

The following abbreviations are used in this manuscript:

D_0	Alginate-based wound dressing used as a control
D_0.1	Alginate-based wound dressing containing 0.1% liposomal curcumin
D_0.2	Alginate-based wound dressing containing 0.2% liposomal curcumin
D_0.3	Alginate-based wound dressing containing 0.3% liposomal curcumin
D_0.4	Alginate-based wound dressing containing 0.4% liposomal curcumin
D_0.5	Alginate-based wound dressing containing 0.5% liposomal curcumin
SE	Serous exudate, superficial wound
IE	Inflammatory exudate, purulent exudate
IASE	Infected exudate with abundant secretion
FTIR	Fourier-transform infrared
WVT	Water-vapor transmission
PT	Prothrombin time
