# Supplementary material for: Development, Characterization, and In Vitro Hemostatic Assessment of an Alginate-Based Wound Dressing Incorporating Liposomal Curcumin
Source: Gels. 2026 Jul 14;12(7):626. doi: 10.3390/gels12070626 (PMC13408368; doi:10.3390/gels12070626)
Supplement: Supplementary file 1 [file gels-12-00626-s001.zip › gels-4422080-supplementary.pdf]

Development, characterization, and *in vitro* hemostatic assessment of an alginate-based wound dressing incorporating liposomal curcumin

Antonela Nagy[1], Leonard Mihaly Cozmuta[1], Melisa Marcus[1], Beatrice Mihalescu[1], Anca Mihaly Cozmuta[1*]

[1] **Technical University of Cluj-Napoca, North University Center of Baia Mare, Victoriei Str. 76, Baia Mare, Romania;** *antonelanagy2@gmail.com.*

[1] **Technical University of Cluj-Napoca, North University Center of Baia Mare, Victoriei Str. 76, Baia Mare, Romania;** *mihalyleonard@yahoo.com.*

[1] **Technical University of Cluj-Napoca, North University Center of Baia Mare, Victoriei Str. 76, Baia Mare, Romania;** *bojemelisa@gmail.com.*

[1] **Technical University of Cluj-Napoca, North University Center of Baia Mare, Victoriei Str. 76, Baia Mare, Romania;** *ancamihalycozmuta@gmail.com.*

[1] **Technical University of Cluj-Napoca, North University Center of Baia Mare, Victoriei Str. 76, Baia Mare, Romania;** *Mihalescu.Va.Beatric@student.utcluj.ro.*

[1] **Technical University of Cluj-Napoca, North University Center of Baia Mare, Victoriei Str. 76, Baia Mare, Romania;** **ancamihalycozmuta@gmail.com*

***Correspondence: ancamihalycozmuta@gmail.com.**

2.AM.Results and discussions

2.1.SM.Chromatic components

Table S1. Chromatic components of alginate-based dressing contain 0.2% liposomal turmeric.

purple<br>380-449 nm	blue<br>450-484 nm	cyan<br>485-499 nm	green<br>500-564 nm	yellow<br>565-589 nm	orange<br>590-624 nm	red<br>625-760 nm
3.87 ± 0.14	4.54 ± 0.12	4.97 ± 0.17	11.26 ± 0.45	23.50 ± 0.94	25.68 ± 0.92	28.39 ± 1.17

The results are presented as mean ± standard deviation (n = 3).

2.2.SM. Thickness

The thickness of the alginate-based dressings was significantly affected by the incorporation of 0.2% turmeric. The control alginate dressing exhibited a thickness of 0.246 ± 0.01 mm, whereas the turmeric-loaded dressing showed an increased thickness of 0.289 ± 0.01 mm. Statistical analysis confirmed that this difference was significant ($p < 0.05$). The observed increase in thickness may be attributed to structural modifications within the alginate matrix induced by the presence of turmeric particles, potentially leading to a more heterogeneous network and increased solid content. Although the absolute change in thickness was relatively small, such variations may still influence the overall physicochemical performance of the dressings, including fluid handling capacity and mechanical behavior.

2.3. SM.Color analysis

The colorimetric properties of the D_0.2 dressing evaluated using the CIE L*a*b* system, yield values of L* = 47.22, a* = 16.68, and b* = 39.53. The relatively low L* value indicates a moderately dark appearance, suggesting substantial incorporation of the turmeric-derived pigment within the polymeric matrix. The positive a* value reflects a noticeable red component, while the high positive b* value confirms a strong yellow contribution. This combination of chromatic coordinates is characteristic of a saturated yellow–orange coloration, consistent with the presence of curcumin. Overall, these results demonstrate effective dispersion and visual manifestation of the encapsulated turmeric within the alginate network, contributing to a homogeneous and intensely colored dressing.

3.SM. Materials and methods

3.1.SM. Thickness characterization

Thickness is a clinically relevant parameter for hydrogel dressings, as it influences both fluid management and patient comfort. Adequate thickness provides sufficient capacity for exudate absorption and retention, helping to maintain a moist wound environment while preventing leakage. At the same time, thickness affects mechanical properties such as flexibility and conformability; overly thick dressings may be less adaptable to irregular wound surfaces, whereas very thin ones may lack

structural integrity and durability. Additionally, thickness can impact oxygen permeability and the diffusion of therapeutic agents. Therefore, optimizing dressing thickness is essential to balance absorbency, mechanical performance, and patient comfort. The thicknesses of hydrogel dressings were measured using a caliper.

3.2.SM. Color analysis

The color of the dressings was evaluated using the CIELab* color space. Measurements were carried out with a YL4560 non-contact bench spectrophotometer (Shenzhen ThreeNH Technology Co., Ltd). Prior to measurement, the instrument was calibrated against standard white and black tiles. For each sample, readings were recorded at five different locations at 25 °C, and the results are reported as mean ± standard deviation.

3.3. Polyphenols release

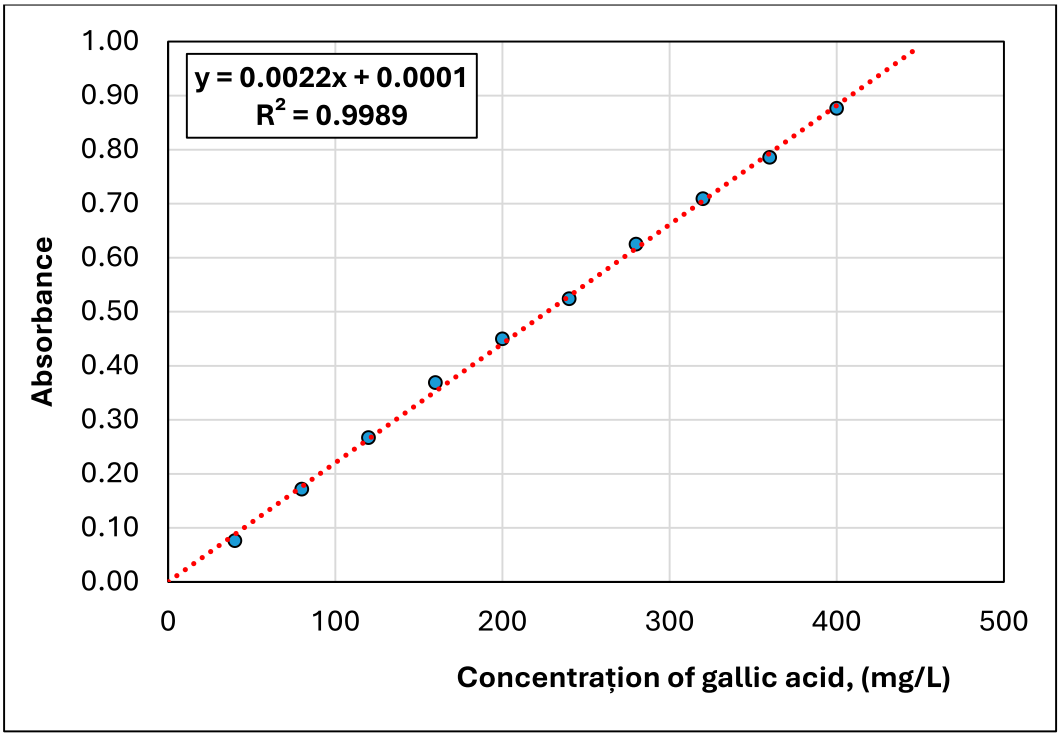

Figure S1. Calibration curve for polyphenols release from dressings
